# Atypical imaging of spinal tuberculosis: a case report and review of literature

**DOI:** 10.11604/pamj.2016.24.101.9701

**Published:** 2016-05-31

**Authors:** Huijun Zhang, Zenghui Lu

**Affiliations:** 1 Department of Orthopedics, Xi'an Chest Hospital, Xi'an, China

**Keywords:** Spinal tuberculosis, imaging, case report

## Abstract

This is a case report of spinal tuberculosis combined with sacroiliac joint tuberculosis, pulmonary tuberculosis, chest wall tuberculosis and tuberculous pleurisy and the image of the patient is rare, special and not typical and it looks like a halo sign. It has an important reference value for the diagnosis of spine tuberculosis although it is a rare imaging manifestation and diagnosis was confirmed by pathology after the surgery. Therefore atypical imaging is often appeared in clinical practice and it is meaningful and necessary for the diagnosis of atypical spinal tuberculosis combined with multiple organ tuberculosis.

## Introduction

Imaging examination is an important method in the diagnosis of spinal tuberculosis and most cases can be diagnosed through typical imagings of spinal tuberculosis as well as clinical manifestations, laboratory examination, medical history. But there is also some images of spinal tuberculosis are special and atypical in clinical. This gives us the opportunity to study and confirm that it is indeed the imaging manifestations of spinal tuberculosis. Rare imaging, such as atypical CT of the spine, Should be able to provide help and reference for the diagnosis of spinal tuberculosis, especially for those suffering from multiple organ tuberculosis patients.

## Patient and observation

**Figure 1:**
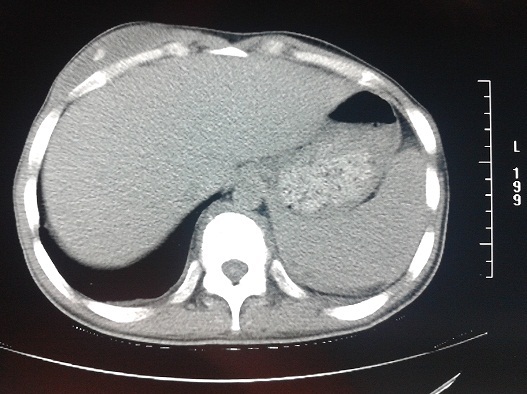
The right chest wall mass with abscess formation and calcified shadow

**Figure 2:**
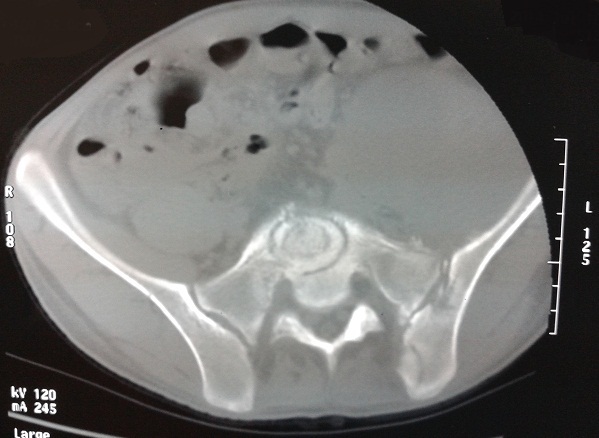
Sacroiliac joint bone destruction and left huge abscess

**Figure 3:**
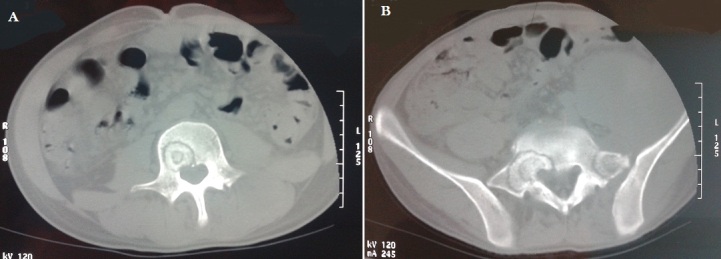
(A,B) the specific performance of lumbar vertebral bone destruction in L4 and L5 and they look like a halo sign

**Figure 4:**
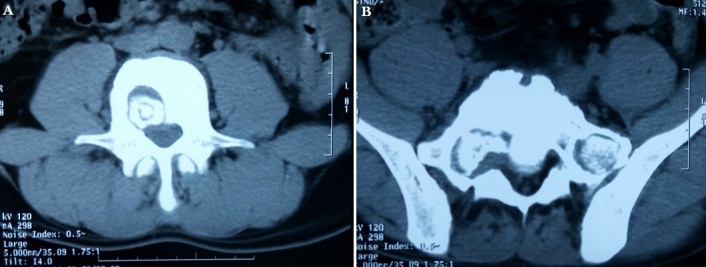
(A,B) the changes of lumbar vertebral bone destruction in L4 and L5 after 15 months of treatment

**Figure 5:**
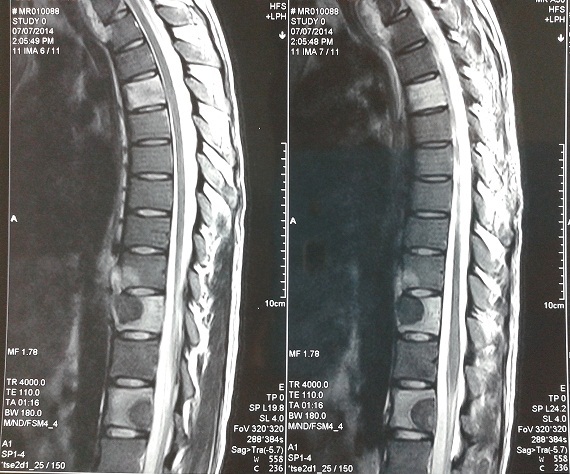
The specific performance of bone destruction in T1, T4, T5, T10 and T12 vertebral bodies and they look like a round of bone destruction

A 22-year-old male patient who was a farmer had a brief-history of waist pain and chest wall mass and the patient was suggested to undergo anti-tuberculosis therapy, which not subsided after a standard 4 week therapy of anti-tuberculosis. He complained of waist pain during the treatment and chest wall mass did not change, for which he was given pain killers and was advised rest. The pain disappeared after the use of pain-killer pills (500mg), but they did not gave total relief. Doctors advised two CT's scan (Computed Tomography Scan) of Chest and Spine and a MRI of Spine. CT's were clear. CT scan of chest shows the right chest wall mass with abscess formation and calcified shadow (Figure 1). CT scan of rib, not done to rule out bone destruction, was maybe abnormal and CT scan of sacroiliac joint shows obvious bone destruction and the huge abscess (Figure 2). Nevertheless, the CT features of lumbar vertebral bone destruction in L4 and L5 is the most impressive and the CT images of the patient are special and not typical, which look like a halo sign (Figure 3 (A,B)). It is very rare CT features of spinal tuberculosis infection, Moreover, CT features of spinal tuberculosis is changing after anti-tuberculosis treatment for more than 1 years later. Another CT Scan done at 15 months revealed the changes of spinal bone destruction in L4 and L5 (Figure 4 (A,B)). It may be related to the patient's own immunity and we need to search the specific reason. MRI of spine shows areas of bone destruction in T1, T4, T5, T10 and T12 vertebral bodies and it looks like a round of bone destruction (Figure 5). It is a difference compared with typical MRI findings of spinal tuberculosis and it is impressive. The patient, who was infected with tuberculosis for multiple organs, was diagnosed as spinal tuberculosis combined with sacroiliac joint tuberculosis, pulmonary tuberculosis, chest wall tuberculosis and tuberculous pleurisy and he needed surgical treatment. Surgery was undertaken when the ESR (erythrocyte sedimentation rate) decreased and after the preoperative examination without surgical contraindications and diagnosis was confirmed by pathology after the surgery. He was advised complete bed rest and was given a lumbosacral brace to be worn and spinal movements were restricted. Multi-vitamin injections were given and a high protein diet was recommended after the operation. Anti-tuberculosis treatment of the patient has been carried out so far after the surgery. Furthermore, the patient with waist pain has disappeared and the body weight increased significantly 1 year later. There was a weight gain of 8 kgs and there were no complications.

## Discussion

In recent years, there has been an increase in the numbers of spinal TB cases and we, therefore, want to report such a case of our patient, who was diagnosed as spinal tuberculosis combined with sacroiliac joint tuberculosis, pulmonary tuberculosis, chest wall tuberculosis and tuberculous pleurisy, whose images are special and not typical and it is rare in clinical. But it is indeed the characteristics of bone destruction in spinal tuberculosis imaging. Spinal TB should always be suspected when radiographs demonstrate a destructive spinal process. Awareness and prompt management of TB spine will help in reducing the continuing morbidity of this disease [1]. Spine tuberculosis results from an infection of the bone caused by the Mycobacterium tuberculosis bacteria via a combination of haematogenous root and lymphatic drainage. TB starts in the lung but it can travel to any organ or structure of the body. The spinal column is often the most affected extra-pulmonary site [2]. Pain, the most common presenting symptom, usually has an insidious onset and it may be mechanical in nature during the initial stages. Persistent spinal pain, local tenderness, limitation of spinal mobility, febrile state and neurologic complications, present as the destruction continues. Other systemic symptoms are reflective of chronic illnesses including weight loss and fatigue [3, 4]. In the reported case, Antituberculosis agents, the standard being a combination of Isoniazid, Rifampicin and Pyrazinamide, with Ethambutol, are essential for management, with chemotherapy, for at least 18 months, which are preferred to shorter courses [5]. And it should be actively implemented no matter whether the operation. Several studies have reported that surgery is the mainstay of treatment and we also take the patient with surgical treatment and we provided a detailed and strict treatment schedule of 18 months, combined with good nutritional support, bed-rest and lumbosacral braces, while ambulant, were the mainstay of management, for complete recovery of patients. Moreover, a prolonged follow-up is essential in case of spinal tuberculosis, as in the presented case.

Imaging examination plays a very important role in diagnosis of spinal tuberculosis and CT examination is the most economical and effective in clinical. Very often, the clinical presentation, together with the radiologic appearance of the spine and a positive tuberculin test, may suggest spinal TB, but the diagnosis must be confirmed by evidence of acid-fast bacilli from the bone or body fluids or postoperative pathology results. In the era of modern imaging modalities and effective anti-TB medication, spinal TB appears to be a disease which has occasionally a few atypical imaging findings in clinical. In any patient who presents with a destructive lesion of the spine, CT or MRI of the spine may allow in making an early diagnosis and evaluation of spine tuberculosis, while a biopsy is essential to confirm the diagnosis of TB, bacteriologically and histologically. Correlative CT and MRI have made diagnosis of spinal TB substantially easier and faster. Major advantage of these studies is their ability to show lytic lesions and adjacent abscess formations [6, 7]. A fragmentary type of bone destruction was the most frequent CT feature of the disease followed by the lytic type intervertebral disc destruction and paravertebral mass/abscess were other features [8]. CT scan was the most useful radiological tool, both for making the diagnosis and for determining the extent of the disease. In our patient, CT scan revealed positive findings such as anterior vertebral body destruction, para-spinal abscesses. This case, therefore, supports a better imaging data of atypical spinal tuberculosis.

## Conclusion

Spinal tuberculosis has a high incidence in China and imaging examination is an important method in the diagnosis of spinal tuberculosis. Rare imaging which is special and atypical, such as atypical CT of the spine, is meaningful and it may provide reference for the diagnosis of atypical spinal tuberculosis, especially for those suffering from multiple organ tuberculosis patients.
